# *Sulfolobus* chromatin proteins modulate strand displacement by DNA polymerase B1

**DOI:** 10.1093/nar/gkt588

**Published:** 2013-07-01

**Authors:** Fei Sun, Li Huang

**Affiliations:** State Key Laboratory of Microbial Resources, Institute of Microbiology, Chinese Academy of Sciences, Beijing 100101, China

## Abstract

Strand displacement by a DNA polymerase serves a key role in Okazaki fragment maturation, which involves displacement of the RNA primer of the preexisting Okazaki fragment into a flap structure, and subsequent flap removal and fragment ligation. We investigated the role of *Sulfolobus* chromatin proteins Sso7d and Cren7 in strand displacement by DNA polymerase B1 (PolB1) from the hyperthermophilic archaeon *Sulfolobus solfataricus*. PolB1 showed a robust strand displacement activity and was capable of synthesizing thousands of nucleotides on a DNA-primed 72-nt single-stranded circular DNA template. This activity was inhibited by both Sso7d and Cren7, which limited the flap length to 3–4 nt at saturating concentrations. However, neither protein inhibited RNA displacement on an RNA-primed single-stranded DNA minicircle by PolB1. Strand displacement remained sensitive to modulation by the chromatin proteins when PolB1 was in association with proliferating cell nuclear antigen. Inhibition of DNA instead of RNA strand displacement by the chromatin proteins is consistent with the finding that double-stranded DNA was more efficiently bound and stabilized than an RNA:DNA duplex by these proteins. Our results suggest that *Sulfolobus* chromatin proteins modulate strand displacement by PolB1, permitting efficient removal of the RNA primer while inhibiting excessive displacement of the newly synthesized DNA strand during Okazaki fragment maturation.

## INTRODUCTION

DNA replication proceeds in a semi-discontinuous manner owing to the antiparallel nature of DNA double strands and the lack of 3′–5′ DNA polymerase in living organisms. In Eukarya, the leading strand is extended continuously by DNA polymerase ε (Pol ε) (1), whereas the lagging strand is synthesized by Pol δ in discrete RNA-primed segments termed Okazaki fragments (2). Synthesis of the lagging strand is completed after these fragments are processed in an event known as Okazaki fragment maturation, which entails removal of RNA primers, gap filling DNA synthesis and subsequent sealing of nicks. Various pathways in Okazaki fragment maturation have been proposed based on genetic and biochemical studies [reviewed in (3)]. Among them, two pathways triggered by Pol δ-mediated strand displacement are considered prevailing. On encountering the 5′-terminus of the downstream segment after filling the gap between the two Okazaki fragments, Pol δ is able to plow into the double-stranded region, displacing the downstream strand into a flap structure (4). Depending on the size of the flap, Okazaki fragment maturation may proceed in either of the two pathways, i.e. the short flap pathway and the long flap pathway. Most flaps generated by Pol δ vary in size but are no longer than 8 nt (5). These flaps are cleaved directly by flap endonuclease 1 (Fen1), a structure-specific endonuclease (6), generating a nick between the two adjacent Okazaki fragments, which is then ligated by DNA ligase 1 (Lig1) (4,5,7). However, a flap may occasionally escape Fen1 cleavage and grow to a length of up to 30 nt (5). In this case, the long flap is stably bound by replication protein A (RPA), the eukaryotic single-stranded DNA (ssDNA)-binding protein (8). Binding by RPA inhibits Fen1 cleavage but stimulates another endonuclease Dna2, which interacts physically and functionally with RPA (9,10). Dna2 is able to reduce the size of the RPA-coated flap such that the remaining flap is no longer bound tightly by RPA and cleaved completely by Fen1 as in the short flap pathway (7,9,11).

Because Archaea resemble Eukarya in DNA replication, the two may use similar pathways in Okazaki fragment maturation. Homologs of Fen1 and Lig1 have been found in Archaea and both have been shown to function in DNA replication (12–20). In the hyperthermophilic archaeon *Sulfolobus solfataricus*, DNA polymerase B1 (PolB1) is believed to be the only replicative DNA polymerase and to operate on both leading and lagging strands (21–23). The process of Okazaki fragment maturation was recently reconstituted *in vitro* with recombinant PolB1, Fen1 and Lig1 from *S. solfataricus* (23), suggesting the presence of the Fen1-mediated short flap pathway in Archaea. However, *Sulfolobus* species do not appear to use the eukaryotic-type long flap pathway because of the apparent lack of a Dna2 homolog in the organisms (24).

All DNA transactions occur on DNA bound and compacted by chromatin proteins *in vivo*. Sul7d and Cren7, two chromatin proteins similar in structure and DNA binding, exist in abundance in *Sulfolobus* (25–27). Sul7d refers to a group of 7 kDa DNA-binding proteins highly conserved in *Sulfolobus* (28). Sul7d proteins, which account for ∼5% of the total cellular protein (25), exist as monomers in solution (28) and bind double-stranded DNA (dsDNA) nonspecifically with a dissociation constant in a micromolar range (29,30) and a binding size of 4–6 base pairs (29,31,32). On binding to dsDNA, these proteins raise the melting temperature (*T*_m_) of the DNA by as much as 33°C (33) and constrain DNA in negative supercoils (25,34,35). Cren7, discovered recently in *Sulfolobus shibatae*, is more widely distributed than Sul7d and exists in all genome-sequenced Crenarchaea except for *Thermophilum pendens* Hrk5 (26). Cren7 from *S. shibatae* is 6.5 kDa in size, monomeric and constitutes 1% of the cellular protein. Like Sul7d, Cren7 is capable of sequence-nonspecific DNA binding, raising the *T*_m_ of dsDNA and constraining negative DNA supercoils (26). Although Sul7d differs from Cren7 in amino acid sequence, the two proteins are similar in structure (26) and capable of inducing a sharp single-step kink in bound dsDNA (27). Recently, a single-molecule study reveals that both Cren7 and Sul7d compact DNA to similar extents by inducing a rigid bend (36). However, subtle differences exist between the two proteins. Cren7 has a larger binding size (8 bp) and constrains negative supercoils more efficiently than Sul7d (26). Structural comparison also reveals differences between Cren7 and Sul7d in DNA binding surface and binding pattern, suggesting that the two proteins serve different roles in chromosomal organization (27,37).

It is of interest to understand how these proteins may influence various fundamental processes involving DNA. In a previous study, Ssh7, an Sul7d protein from *S. shibatae*, was shown to be able to enhance the polymerization activity while inhibiting the exonuclease activity of PolB1 without affecting its proofreading ability (38). How Sul7d and Cren7 may affect Okazaki fragment maturation, a critical step in the lagging strand synthesis, has yet to be investigated. Here, we show that both Sul7d and Cren7 are capable of inhibiting the robust strand displacement activity of PolB1, reducing the size of the displaced DNA strand to 3–4 nt. However, the ability of PolB1 to displace RNA primers was not affected by either protein. Our data suggest that chromatin proteins play a key role in Okazaki fragment maturation in Crenarchaea.

## MATERIALS AND METHODS

### Proteins

Recombinant *S. solfataricus* PolB1, proliferating cell nuclear antigen (PCNA: PCNA1/PCNA2/PCNA3), replication factor C (RFC: RFC_S_/RFC_L_), Sso7d and Cren7 were prepared as described previously (12,26,38). Protein concentrations were determined by the Lowry method using bovine serum albumin (BSA) as the standard (39).

### Substrates

DNA oligonucleotides were synthesized at Sango BioTech (Shanghai, China). RNA and RNA–DNA chimeric oligonucleotides were synthesized at Takara (Dalian, China). Sequences of the oligonucleotides are shown in Supplementary Table S1. Minicircular ssDNA (C72) was prepared as described previously (4) with modifications. A 72-nt 5′-phosphorylated linear ssDNA (L72) (1 nmol) was mixed with a 39-nt bridger ssDNA (1.5 nmol), which was complementary to the 15-nt region at the 5′-end and the 24-nt region at the 3′-end of L72, in 50 mM Tris–HCl, pH 8.0, 10 mM MgCl_2_, 10 mM dithiothreitol (DTT), 1 mM ATP, 0.025 mg/ml BSA in a total volume of 12 ml. The mixture was incubated for 10 min at 75°C and gradually cooled down to room temperature so that the two ends of L72 were brought together by annealing to the bridger. T4 DNA ligase (120 U, New England Biolabs) was added, and ligation was carried out for overnight at 16°C. The sample was extracted with phenol/chloroform and the DNA was precipitated with ethanol. The DNA was dissolved in Tris-EDTA (TE) buffer (10 mM Tris–HCl, pH 8.0, 1 mM EDTA) and loaded onto an 8% denaturing polyacrylamide gel containing 7 M urea. After electrophoresis, the gel was stained with ethidium bromide and the band corresponding to C72 was sliced under UV light. The gel slice was crushed and soaked overnight at 37°C in a solution containing 0.5 M ammonium acetate, 100 mM magnesium acetate, 1 mM EDTA, pH 8.0, and 0.1% sodium dodecyl sulphate (SDS). The supernatant was collected, and the DNA was precipitated twice with ethanol and dissolved in TE. The product was confirmed cyclic because no degradation was observed when treated with Exonuclease I (New England Biolabs).

To prepare a primer template, a primer was labeled at the 5′-end using T4 polynucleotide kinase (Takara) and [γ-^32^P]ATP (PerkinElmer), and the labeled primer was purified using a G25 microspin column (GE Healthcare). The labeled primer was annealed to L72 or C72 at a molar ratio of 1:1.5 to ensure complete hybridization of the primer to the template. Annealing reactions were carried out in 20 mM Tris–HCl, pH 8.0, and 100 mM NaCl.

### PolB1 assays

The standard reaction (20 μl) contained 2 nM primer template, 20 nM PolB1, indicated amounts of Sso7d or Cren7, 50 mM Tris–HCl, pH 8.0, 2 mM β-mercaptoethanol, 0.1 mg/ml BSA, 4 mM MgCl_2_ and 1 mM dNTPs (Roche). The mixture was incubated at 65°C for 15 min unless specified. For reactions containing PCNA (PCNA1/PCNA2/PCNA3) and RFC (RFC_S_/RFC_L_), 100 nM PCNA and 100 nM RFC as well as 1 mM ATP were preincubated with the primer template for 5 min at 70°C to load PCNA onto the circular template. After the addition of indicated amounts of a chromatin protein and 5 nM PolB1, the mixture was incubated at 70°C for another 15 min. The reaction containing no chromatin protein was quenched by an equal volume of 2× loading buffer [95% deionized formamide, 100 mM EDTA, 0.025% bromphenol blue and 0.025% xylene cyanol FF for denaturing polyacrylamide gel electrophoresis (PAGE) or 100 mM NaOH, 10 mM EDTA, 6% Ficoll-400, 0.025% bromocresol green and 0.025% xylene cyanol FF for alkaline agarose gel electrophoresis]. When a chromatin protein was included in the reaction, a solution (4 μl) containing 3% SDS, 150 mM EDTA and 15 mg/ml proteinase K (Promega) was added to the sample. The mixture was incubated for 45 min at 50°C to digest the chromatin protein and extracted with phenol/chloroform. The supernatant was then mixed with the loading buffer. After boiling for 3 min and subsequent cooling on ice, the sample was subjected to electrophoresis either in 8% denaturing polyacrylamide gel containing 7 M urea in 1× Tris borate-EDTA (TBE) or in 1.2% alkaline agarose gel in 50 mM NaOH and 1 mM EDTA. For sequencing gel electrophoresis, the sample was concentrated by ethanol precipitation, dissolved in the loading buffer and resolved by 8% denaturing PAGE containing 7 M urea in 1× TBE. The gel was dried and exposed to radiographic film.

### Electrophoretic mobility shift assays

A ^32^P-labeled oligonucleotide (D30 or R30) was annealed to its complementary ssDNA (Com30) at a molar ratio of 1:1.5, yielding a 30-bp dsDNA or 30-bp RNA:DNA hybrid fragment. Various amounts of Sso7d or Cren7 was incubated with the radiolabeled nucleic acid fragment (2 nM) for 10 min at room temperature in 50 mM Tris–HCl, pH 8.0, 2 mM β-mercaptoethanol, 0.1 mg/ml BSA, 4 mM MgCl_2_ and 5% glycerol (v/v). The protein–nucleic acid complexes were electrophoresed in 5% polyacrylamide gel in 0.1× TBE. Following electrophoresis, the gel was dried and exposed to radiographic film or analyzed using an ImageQuant Storm PhosphorImager (GE Healthcare).

### Thermal denaturation of dsDNA and RNA:DNA hybrid

Recombinant Sso7d or Cren7 was mixed with a 15-bp dsDNA, prepared by annealing D15 with Com15, or an RNA:DNA hybrid, made by annealing R15 with Com15, in 10 mM potassium phosphate buffer, pH 7.0. Thermal denaturation of the nucleic acids was measured at A_260_ in a temperature range from 20° to 90°C using a DU800 UV/Visible spectrophotometer (Beckman). The melting temperature was calculated using the DU800 software.

## RESULTS

### PolB1 is capable of efficient strand displacement at high temperature

**Figure 1.**
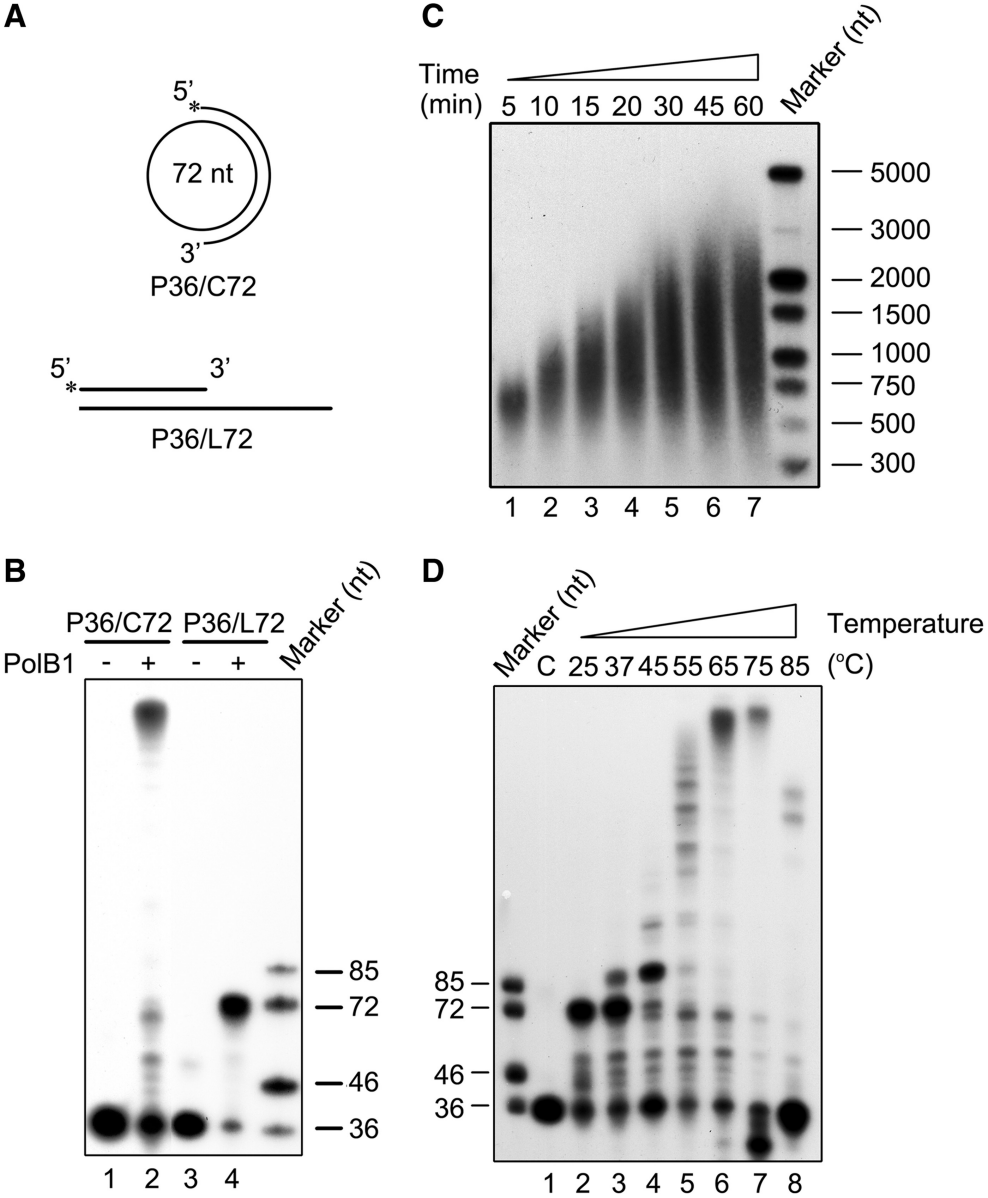
DNA strand displacement by PolB1. (**A**) Schematic representation of the circular and linear primer templates used in PolB1 assays. (**B**) Strand extension by PolB1. PolB1 (20 nM) was incubated for 15 min at 65°C with P36/C72 or P36/L72 (2 nM) under the standard assay conditions. Products were subjected to electrophoresis in 8% polyacrylamide gel containing 7 M urea in 1× TBE. (**C**) Sizes of the products of strand displacement by PolB1. PolB1 (20 nM) was incubated at 65°C for various lengths of time with P36/C72 (2 nM). Products were resolved in a 1.2% akaline agarose gel in 50 mM NaOH and 1 mM EDTA. (**D**) Effect of temperature on strand displacement by PolB1. PolB1 (20 nM) was incubated for 15 min at indicated temperatures with P36/C72 (2 nM). Products were subjected to electrophoresis in 8% polyacrylamide gel containing 7 M urea in 1× TBE. (C) no PolB1 was added. Gels were dried and exposed to radiographic film.

Displacement of the downstream strand annealed to the template into a flap structure represents the first step in Okazaki fragment maturation. So we first investigated the strand displacement activity of PolB1 from *S. solfataricus*. A 72-nt circular ssDNA (C72) was annealed to a 36-nt ssDNA (P36) labeled at the 5′-end, producing P36/C72 (Figure 1A), the primer template used in our strand displacement assays. As shown in Figure 1B, PolB1 synthesized products much longer than 72 nt on the circular template, indicating that the polymerase displaced the primer P36 and the newly synthesized strand after gap-filling synthesis. The long products apparently did not result from terminal transfer by PolB1, which was reported to possess an efficient terminal transferase activity (40) because a product obtained on a linear template L72 was 72 nt in length under our assay conditions in a control experiment (Figure 1A and B).

To learn more about the ability of PolB1 to displace a templated DNA strand, we performed a time course analysis of the products generated by the enzyme on P36/C72. PolB1 synthesized products of ∼750 and ∼3000 nt in size, or over 10 and 40 times longer than the minicircle, after incubation for 5 and 60 min, respectively (Figure 1C). These observations indicate that PolB1 has a robust strand displacement activity.

Because *Sulfolobus* lives optimally at ∼80°C, we determined the effect of temperature on strand displacement by PolB1 on P36/C72. As shown in Figure 1D, PolB1 extended the primer to 72 nt, but produced no longer products at 25°C, suggesting that PolB1 lost the strand displacement ability while retaining the polymerization activity at the low temperature. Strand displacement was detected at 37°C and considerably enhanced at 55°C. Optimal strand displacement occurred at 65°C, where products of ∼1500 nt in size were synthesized (Figure 1C). A further increase in temperature to 75 or 85°C was accompanied by a reduction in the amounts of extension products, presumably due to denaturation of the primer template (*T*_m_ = 65.8°C) at the temperatures. Generation of products shorter than the input primer at 75°C appears to have resulted from cleavage of the denatured primer by the 3′–5′ exonuclease activity of PolB1. Less primer cleavage occurred at 85°C than at 75°C, probably because the exonuclease activity of PolB1 was inhibited at the higher temperature (41). It was previously reported that *S. solfataricus* PolB1 was able to displace only 7–10 nt of the downstream strand on a linear template (23). This low efficiency in strand displacement might be attributed to the fact that the reactions were performed at 50°C, or 15°C below the temperature optimal for strand displacement by PolB1, in that study.

### Sso7d inhibits DNA strand displacement by PolB1

**Figure 2.**
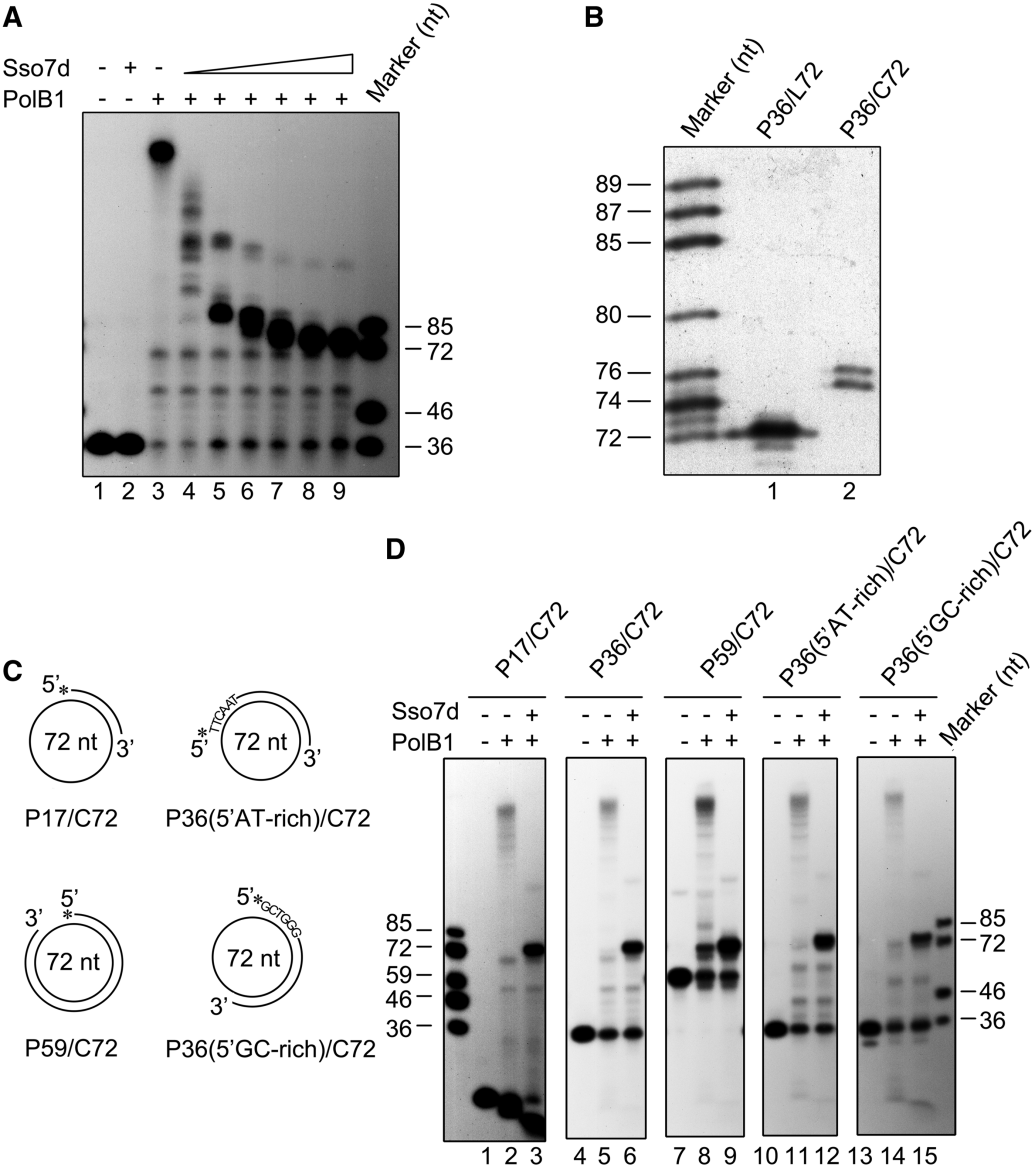
Inhibition of PolB1-mediated DNA strand displacement by Sso7d. (**A**) Effect of Sso7d on strand displacement by PolB1. PolB1 (20 nM) was incubated for 15 min at 65°C with P36/C72 (2 nM) in the presence of various amounts of Sso7d. The reaction mixtures were treated with proteinase K and extracted with phenol/chloroform. Samples were subjected to electrophoresis in 8% polyacrylamide gel containing 7 M urea in 1× TBE. Lane 1, control; lane 2, 90 μM Sso7d; lanes 3–9, Sso7d was added to 0, 0.5, 2.5, 10, 25, 50 and 90 μM, respectively. (**B**) Sizes of the products of strand displacement by PolB1 on templates bound maximally by Sso7d. PolB1 (20 nM) was incubated for 15 min at 65°C with P36/C72 or P36/L72 (2 nM) in the presence of 90 μM Sso7d. Reaction products were subjected to electrophoresis in an 8% sequencing gel in 1× TBE. (**C**) Sketches of primer templates used in experiments shown in Figure 2D. Primers P17 and P59 are annealed to C72 starting from the same base on the minicircle as that for P36. P36(5′AT-rich) and P36(5′GC-rich), which carry 5′-AT-rich and 5′-GC-rich sequences, respectively, are annealed to different regions of C72. (**D**) Template dependence of the inhibition of strand displacement by Sso7d. PolB1 (20 nM) was incubated for 15 min at 65°C with an indicated primer template (2 nM) in the presence or absence of Sso7d (90 μM). Reaction products were subjected to electrophoresis in 8% polyacrylamide gel containing 7 M urea in 1× TBE. Gels were dried and exposed to radiographic film.

To understand the potential role of chromatin proteins in Okazaki fragment maturation, we first examined the effect of Sso7d on the ability of PolB1 to displace the downstream DNA strand on primer template P36/C72. As shown in Figure 2A, when Sso7d was titrated into the PolB1 strand displacement reaction, the size of the extension product decreased with an increasing Sso7d concentration, demonstrating the inhibitory effect of the chromatin protein on DNA strand displacement by PolB1. However, gap-filling synthesis by PolB1 on the template was not influenced by Sso7d. Intriguingly, extension products were longer than 72 nt even when Sso7d was added to 90 μM, a concentration far exceeding that required to bind maximally the double-stranded products of the reaction (Figure 2A). Sequencing gel analysis revealed that these products were 75–76 nt in length. By comparison, the product synthesized by PolB1 on P36/L72 was exactly 72 nt in size (Figure 2B). Therefore, PolB1 was able to displace only a short DNA strand of 3–4 nt in length in the presence of Sso7d at a saturating level, i.e. the level required for maximal inhibition of strand displacement.

We then tested if template primers of different sequences or sizes would affect the inhibition of PolB1-mediated DNA strand displacement by Sso7d. We found that PolB1 efficiently displaced each of the five tested primers differing in size or sequence, which were annealed to C72, in the absence of Sso7d (Figure 2C and D). The addition of Sso7d (90 μM) restricted strand displacement to 3–4 nt, yielding products of the same size. Primer cleavage occurred when P17/C72 was used in the assays, presumably because this primer template, which had a relatively short double-stranded region, was more readily denatured and cleaved by PolB1 than other primer templates containing a longer double-stranded region at the assay temperature. Our results indicate that Sso7d is a potent inhibitor of DNA strand displacement by PolB1.

### Stand displacement by PolB1 is not blocked by a short 5′-end flap of the downstream strand of Sso7d-bound templates

**Figure 3.**
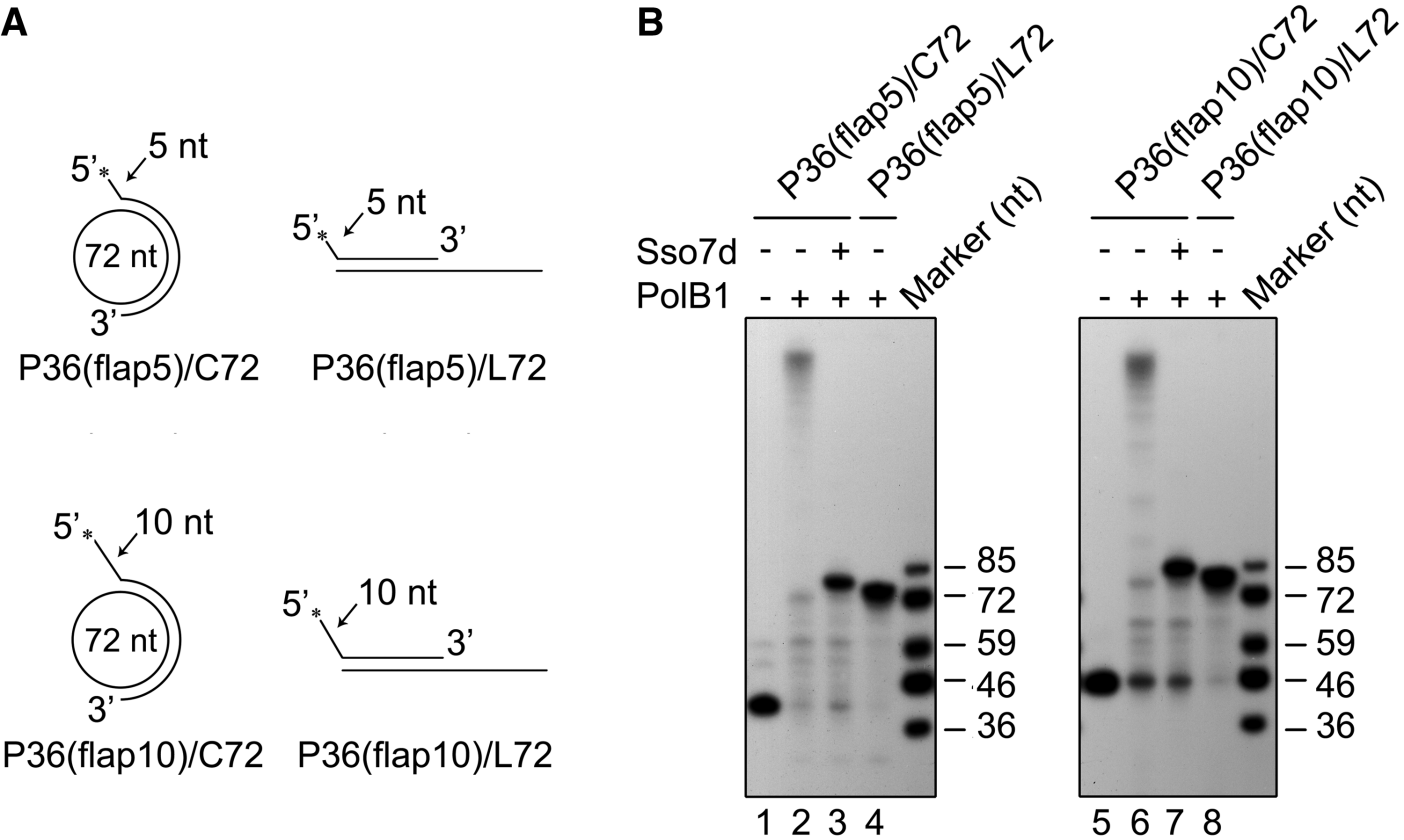
Stand displacement by PolB1 is not blocked by a 5′-end flap on an Sso7d-bound template. (**A**) Schematic representation of primer templates used in experiments shown in Figure 3B. (**B**) Strand displacement by PolB1 on a primer template containing a flap of 5 or 10 nt at the 5′-end of the primer in the presence of saturating amounts of Sso7d. PolB1 (20 nM) was incubated with an indicated primer template (2 nM) in the presence or absence of Sso7d (90 μM). Reaction products were subjected to electrophoresis in 8% polyacrylamide gel containing 7 M urea in 1× TBE. Gels were dried and exposed to radiographic film.

The observation that PolB1 was able to displace a short stretch of 3–4 nt on P36/C72 in the presence of Sso7d at saturation raised a possibility that strand displacement by the polymerase stopped only when encountering a short flap. It was reported that a ‘strand opening’ step existed in Pol δ-mediated strand displacement, where Pol δ occasionally paused after 1–5 nucleotides were displaced (42,43). It may be speculated that PolB1-mediated strand displacement is preceded by a strand opening step involving the formation of a short flap structure, and the chromatin protein functions at this step, preventing the translocation of the polymerase further along the template. To test this possibility, two primers, which, when annealed to C72 or L72, formed a 5 - and 10-nt flap at the 5′-end of the primer, were synthesized (Figure 3A). If the hypothesis holds, strand displacement would no longer take place when PolB1 runs into the preformed flap and the products generated on both the circular and the linear templates would be identical in size. However, PolB1 still synthesized longer products on the minicircle than on the linear template in the presence of Sso7d (Figure 3B). The differences in size between the products obtained on the minicircular template and those on the linear template correspond to the displacement of a short stretch of DNA by PolB1 on the former template, indicating that a flap was not responsible for the termination of strand displacement by PolB1. The strand opening step, presumably accompanied by PolB1 pausing, is not inhibited by Sso7d, whereas the further strand displacement is.

### Sso7d is unable to inhibit RNA strand displacement by PolB1

**Figure 4.**
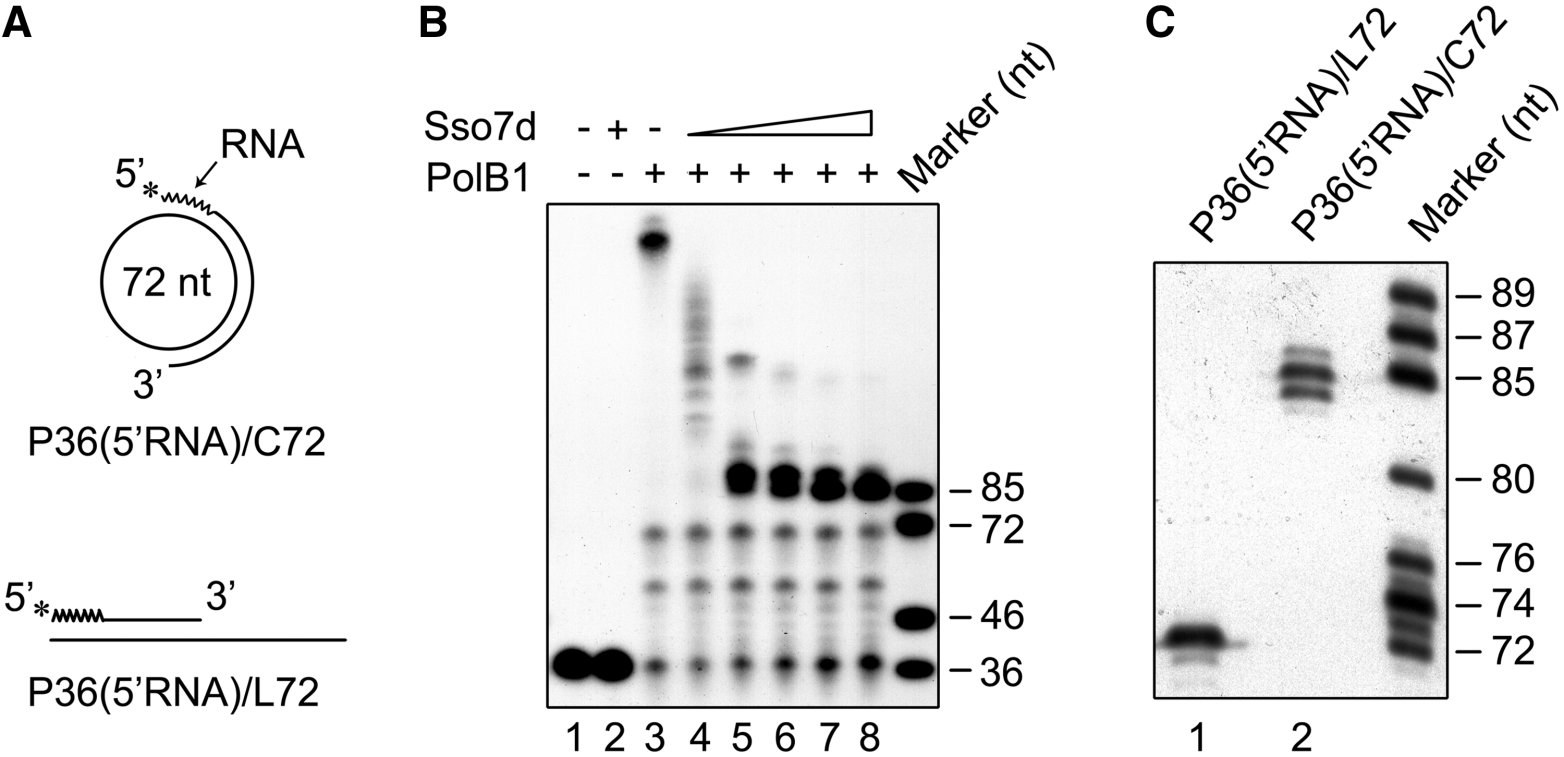
Effect of Sso7d on RNA strand displacement by PolB1. (**A**) Sketches of RNA-primed templates used in experiments shown in Figure 4B and C. The 12-nt oligoribonucleotide portion of the primer was indicated with a wavy line. (**B**) RNA strand displacement by PolB1 on P36(5′RNA)/C72 in the presence of Sso7d. PolB1 (20 nM) was incubated for 15 min at 65°C with P36(5′RNA)/C72 (2 nM) in the presence of various amounts of Sso7d. Lane 1, control; lane 2, 90 μM Sso7d; lanes 3–8, Sso7d was added to 0, 0.5, 2.5, 10, 25 and 90 μM, respectively. Samples were treated with proteinase K and extracted with phenol/chloroform. Reaction products were subjected to electrophoresis in 8% polyacrylamide gel containing 7 M urea in 1× TBE. (**C**) Sizes of the products of strand displacement by PolB1 in the presence of 90 μM Sso7d. PolB1 (20 nM) was incubated for 15 min at 65°C with an indicated template (2 nM) in the presence of Sso7d (90 μM) under the standard assay conditions. Reaction products were subjected to electrophoresis in an 8% sequencing gel in 1× TBE. Gels were dried and exposed to radiographic film.

A replicative DNA polymerase encounters first the downstream RNA strand at the 5′-end of an Okazaki fragment during lagging strand synthesis. We investigated the ability of PolB1 to displace an RNA strand annealed to the minicircular ssDNA using primer template P36(5′RNA)/C72, which was identical to P36/C72 except for the replacement of the first 12 nucleotides at the 5′-end of the primer by a stretch of RNA of the same size (Figure 4A). As shown in Figure 4B, PolB1 displaced the RNA strand as efficiently as it did with the DNA strand. Furthermore, the strand displacement activity of the polymerase on P36(5′RNA)/C72 was also strongly inhibited by Sso7d. Interestingly, however, in the presence of saturating amounts of Sso7d, the sizes of the extension products were significantly longer than those obtained on P36/C72. Further analysis by sequencing gel electrophoresis showed that these products ranged from 84 to 86 nt in length and the 85-nt band was predominant (Figure 4C). The size difference between the major extension product obtained on P36(5′RNA)/C72 and that on P36(5′RNA)/L72 indicates that PolB1 displaced a stretch of 13 nt, which included the entire RNA portion and the first deoxyribonucleotide in the DNA portion of the chimera primer, in the presence of Sso7d at a saturating concentration. Based on these results, we conclude that PolB1 is capable of displacing an RNA strand annealed to a DNA template even in the presence of saturating amounts of Sso7d.

### PCNA/RFC enhances strand displacement synthesis by PolB1 but does not affect the modulation of the activity by Sso7d

**Figure 5.**
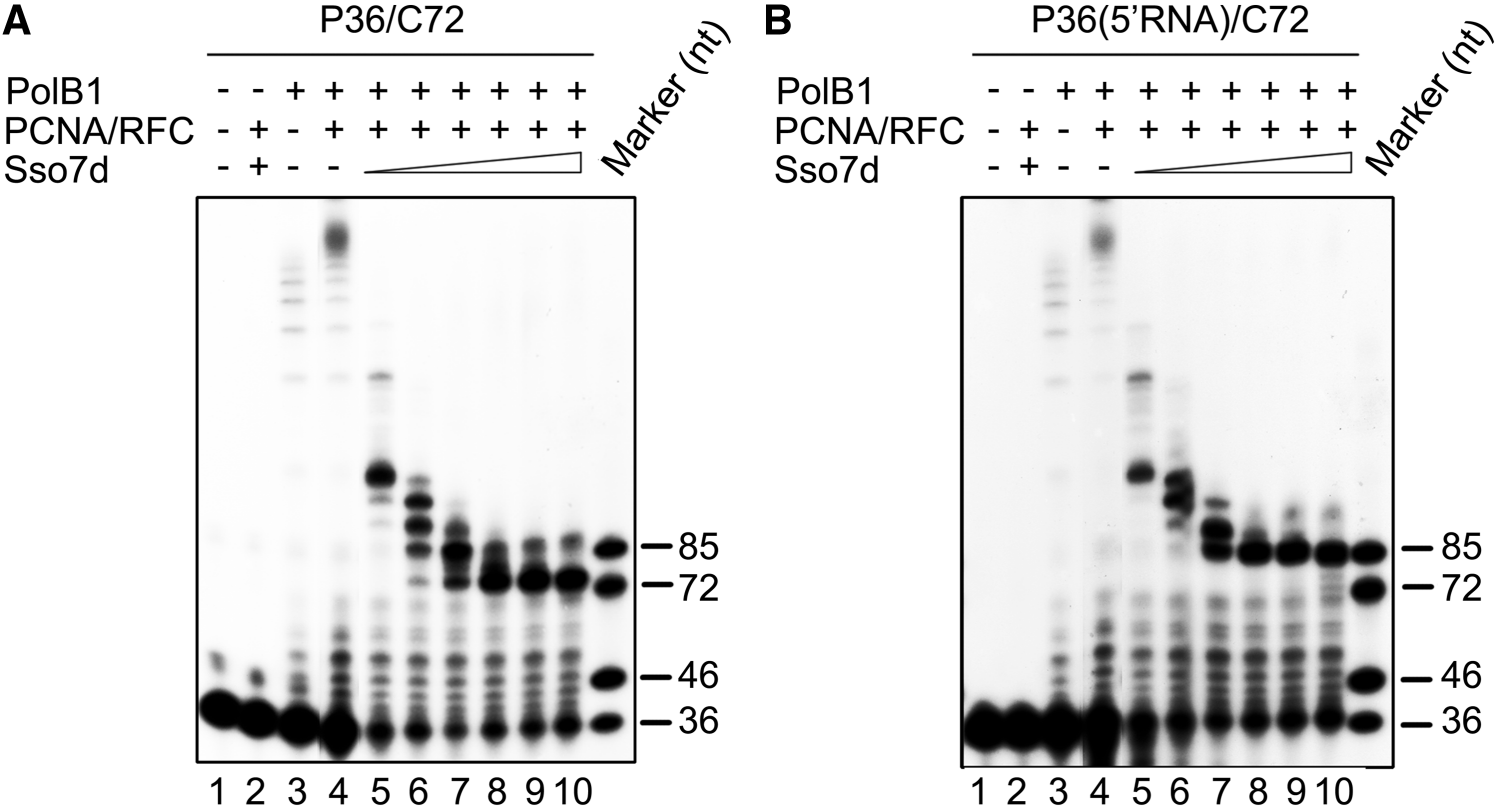
Modulation of PolB1-mediated strand displacement by Sso7d in the presence of PCNA and RFC. (**A**) PolB1-mediated DNA strand displacement. PCNA (100 nM) and RFC (100 nM) were preincubated for 5 min at 70°C with P36/C72. PolB1 (5 nM) and various amounts of Sso7d were added. After 15 min at 70°C, the mixture was treated with proteinase K and extracted with phenol/chloroform. Reaction products were subjected to electrophoresis in 8% polyacrylamide gel containing 7 M urea in 1× TBE. Lanes 5–10, Sso7d concentrations were 0.5, 2.5, 10, 25, 50, 90 μM, respectively. (**B**) PolB1-mediated RNA strand displacement. Reactions were assembled and processed as described in (A) except that P36(5′RNA)/C72, instead of P36/C72, was used as the primer template.

In *S. solfataricus*, the heterotrimeric sliding clamp PCNA, loaded onto a DNA template by the clamp loader RFC in an ATP-dependent manner, tethers PolB1 to the template, enhancing the processivity of the polymerase (12). To learn if strand displacement by PolB1 in association with PCNA was sensitive to inhibition by the chromatin protein, we included PCNA and RFC in the PolB1 strand displacement assays in the presence or absence of Sso7d. To better observe the stimulating effect of PCNA on the activity of PolB1, we reduced the PolB1 concentration to 5 nM in the assays, as compared with 20 nM in the previous assays, and raised the assay temperature to 70°C, at which PCNA loading was most efficient (12). As shown in Figure 5, significantly more strand displacement synthesis by PolB1 on either P36/C72 or P36(5′RNA)/C72 occurred in the presence of PCNA and RFC than in their absence, in agreement with a previous observation (23). However, when Sso7d was titrated into the reaction, strand displacement on both templates was inhibited in similar manners to that in the absence of PCNA and RFC (Figures 2A and 4B). More significant primer extension was observed in the presence of Sso7d than in its absence. This is consistent with the finding that the chromatin protein is capable of enhancing the extension of matched template primers by PolB1 (38). These results indicate that, although the sliding clamp enhances the ability of PolB1 to catalyze strand displacement synthesis, it does not affect the modulation of PolB1-mediated strand displacement by Sso7d.

### Sso7d binds and stabilizes DNA–DNA duplex more efficiently than RNA–DNA hybrid

**Figure 6.**
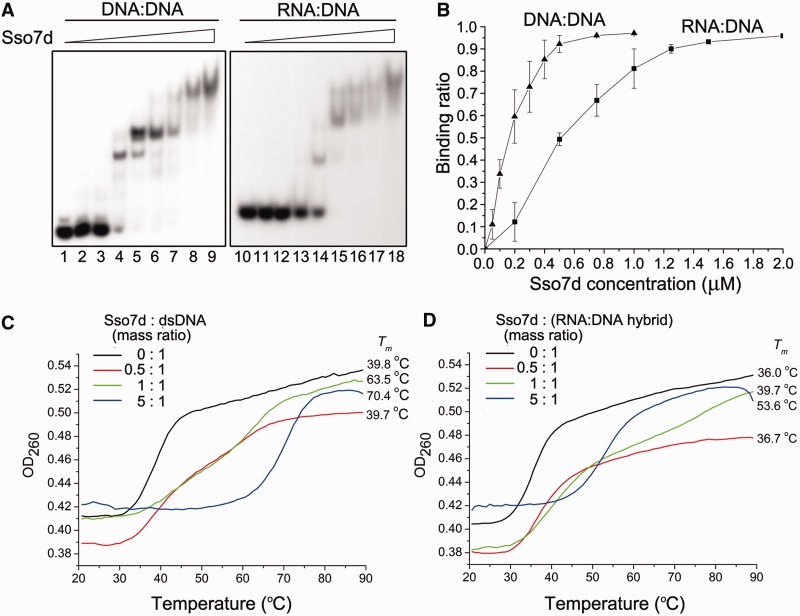
Binding and thermostabilization of a dsDNA fragment and an RNA:DNA hybrid by Sso7d. (**A**) Binding of Sso7d to a dsDNA fragment and an RNA:DNA hybrid. Sso7d was incubated for 10 min at 25°C with a radiolabeled 30-bp dsDNA fragment or a 30-bp RNA:DNA hybrid (2 nM). Samples were loaded onto a 5% polyacrylamide gel and electrophoresed in 0.1× TBE. Gels were dried and exposed to radiographic film. Sso7d concentrations were 0, 0.05, 0.1, 0.5, 1, 5, 10, 50 and 90 μM, respectively. (**B**) A plot of the Sso7d-bound fraction of the radiolabeled probe versus input Sso7d concentration. (**C** and **D**) Effect of Sso7d on the thermal stability of a dsDNA fragment and an RNA:DNA hybrid. Thermal denaturation of a 15-bp dsDNA fragment or an RNA:DNA hybrid in the presence of various amounts of Sso7d was determined by monitoring changes in UV absorbance at 260 nm.

To understand why Sso7d was able to inhibit the displacement of a DNA strand but not an RNA strand from a DNA template by PolB1, we compared the binding affinity of Sso7d for a dsDNA fragment with that for an RNA:DNA hybrid. As revealed by electrophoretic mobility shift assays (EMSA), Sso7d bound the DNA duplex more strongly than the RNA:DNA hybrid, in which the DNA strand was identical to one of the two strands in the DNA duplex, with apparent dissociation constants of 0.15 μM for the DNA duplex and 0.5 μM for the RNA:DNA hybrid (Figure 6A and B). Despite its lower affinity for the RNA:DNA hybrid than that for the DNA duplex, Sso7d was able to bind the hybrid to saturation at high concentrations (e.g. 90 μM) (Figure 6B). However, RNA displacement by PolB1 was not inhibited by Sso7d even at saturating concentrations (Figure 4), suggesting that complete coating of the template by Sso7d per se was insufficient for the inhibition of strand displacement.

Sso7d is known for its ability to stabilize dsDNA by raising its melting temperature (33). However, the effect of Sso7d on the stability of RNA:DNA hybrids was unknown, so we measured the effect of Sso7d on the melting temperatures of both the DNA duplex and the RNA:DNA hybrid. As expected, Sso7d raised the *T*_m_ of the dsDNA by as much as 30.6°C at the protein/DNA mass ratio of 5 (Figure 6C). By contrast, the *T*_m_ of the RNA:DNA hybrid was increased by 17.6°C under the same conditions (Figure 6D). It appears that Sso7d is more effective in stabilizing dsDNA than an RNA:DNA hybrid, consistent with its binding preference for the former over the latter. Because the *T*_m_ of the RNA:DNA hybrid was not as effectively raised by Sso7d as the DNA duplex, a question may arise as to whether the observed inability of Sso7d to inhibit RNA strand displacement was related to the assay temperature. However, this possibility was ruled out because PolB1 still displaced the RNA primer in the presence of Sso7d at high concentrations at 40°C, a temperature close to the *T*_m_ of the RNA:DNA hybrid (data not shown). Taken together, our results suggest that base pairing between two DNA strands and that between a DNA and an RNA strand are differentially stabilized by Sso7d binding such that RNA strand displacement, but not DNA strand displacement, by PolB1 may take place in the presence of the chromatin protein, in agreement with the requirement for processing of the Okazaki fragments.

### Cren7 resembles Sso7d in modulating the strand displacement activity of PolB1

**Figure 7.**
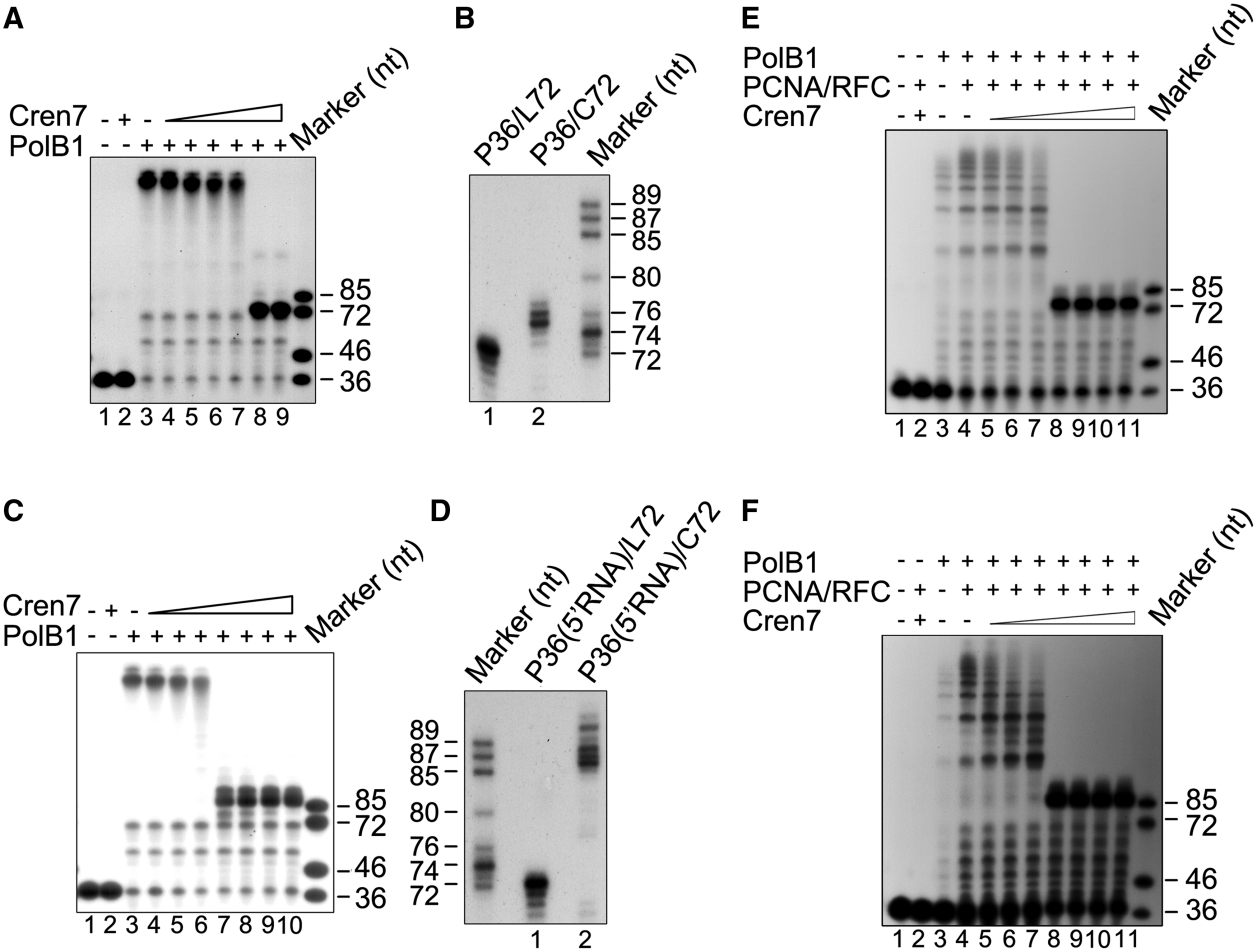
Comparison between Cren7 and Sso7d in modulating strand displacement by PolB1. (**A**) Effect of Cren7 on DNA strand displacement by PolB1. PolB1 (20 nM) was incubated for 15 min at 65°C with P36/C72 (2 nM) in the presence of various amounts of Cren7. Samples were treated with proteinase K and extracted with phenol/chloroform. Reaction products were subjected to electrophoresis in 8% polyacrylamide gel containing 7 M urea in 1× TBE. Lane 1, control; lane 2, 0.64 μM Cren7; lanes 3–9, Cren7 was added to 0, 0.04, 0.08, 0.12, 0.16, 0.32 and 0.64 μM, respectively. (**B**) Sizes of the products of strand displacement by PolB1 on P36/L72 and P36/C72 in the presence of saturating Cren7. PolB1 (20 nM) was incubated with P36/L72 or P36/C72 (2 nM) in the presence of 0.64 μM Cren7 under the standard assay conditions. Reaction products were resolved in an 8% sequencing gel in 1× TBE. (**C**) Effect of Cren7 on RNA strand displacement by PolB1. PolB1 (20 nM) was incubated for 15 min at 65°C with P36(5′RNA)/C72 (2 nM) in the presence of various amounts of Cren7. Lane 1, control; lane 2, 2.5 μM Cren7; lanes 3–10, Cren7 was added to 0, 0.04, 0.08, 0.16, 0.32, 0.64, 1.25 and 2.5 μM, respectively. Reaction products were subjected to electrophoresis in 8% polyacrylamide gel containing 7 M urea in 1 × TBE. (**D**) Sizes of the products of strand displacement by PolB1 on P36(5′RNA)/L72 and P36(5′RNA)/C72 in the presence of saturating Cren7. PolB1 (20 nM) was incubated for 15 min at 65°C with P36(5′RNA)/L72 or P36(5′RNA)/C72 (2 nM) in the presence of Cren7 (2.5 μM) under the standard assay conditions. Reaction products were resolved in an 8% sequencing gel in 1 × TBE. (**E**) Modulation of PolB1-mediated DNA strand displacement by Cren7 in the presence of PCNA and RFC. PCNA (100 nM) and RFC (100 nM) were preincubated for 5 min at 70°C with P36/C72. PolB1 (5 nM) and various amounts of Cren7 were added. After 15 min at 70°C, the mixture was treated with proteinase K and extracted with phenol/chloroform. Reaction products were subjected to electrophoresis in 8% polyacrylamide gel containing 7 M urea in 1× TBE. Lanes 5–11, Cren7 concentrations were 0.04, 0.08, 0.16, 0.32, 0.64, 1.25 and 2.5 μM, respectively. (**F**) Modulation of PolB1-mediated RNA strand displacement by Cren7 in the presence of PCNA and RFC. Reactions were assembled and processed as described in (E) except that P36(5′RNA)/C72, instead of P36/C72, was used as the primer template.

**Figure 8.**
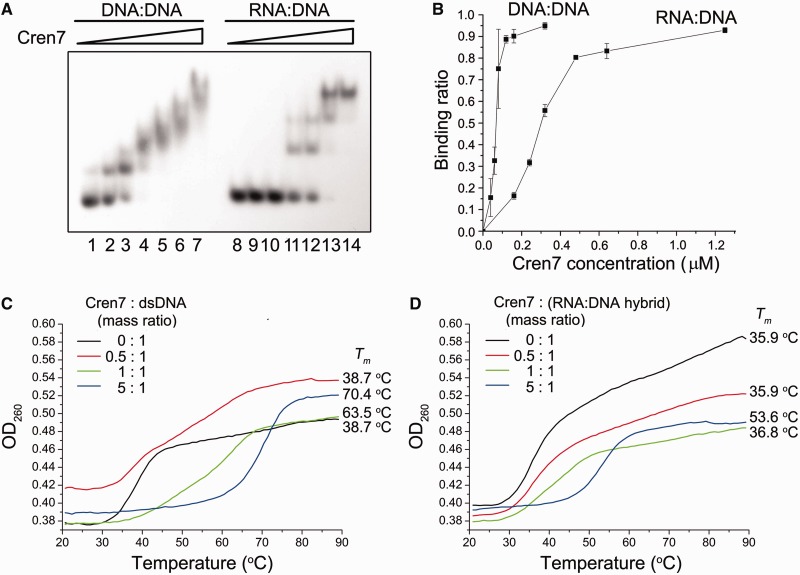
Binding and thermostabilization of a dsDNA fragment and an RNA:DNA hybrid by Cren7. (**A**) Binding of Cren7 to a dsDNA fragment and an RNA: DNA hybrid. Cren7 was incubated for 10 min at 25°C with a radiolabeled 30-bp dsDNA fragment or a 30-bp RNA:DNA hybrid (2 nM). Samples were loaded onto a 5% polyacrylamide gel and electrophoresed in 0.1 × TBE. Cren7 concentrations were 0, 0.04, 0.16, 0.32, 0.64, 1.25 and 5 μM, respectively. Gels were dried and exposed to radiographic film. (**B**) A plot of the Cren7-bound fraction of the radiolabeled probe versus input Cren7 concentration. (**C** and **D**) Effect of Cren7 on the thermal stability of a dsDNA fragment and an RNA:DNA hybrid. Thermal denaturation of a 15-bp dsDNA fragment or an RNA:DNA hybrid in the presence of various amounts of Cren7 was determined by monitoring changes in UV absorbance at 260 nm.

More recently, *S. solfataricus* was found to encode Cren7, another chromatin protein, in addition to Sso7d (26). Cren7, highly conserved among Crenarchaea, resembles Sso7d in structure and biochemical properties including interactions with DNA. In this study, we tested if Cren7 affected strand displacement by PolB1. We found that, like Sso7d, Cren7 also inhibited strand displacement by PolB1, with the length of the single-stranded flap also limited to 3–4 nt in the presence of saturating amounts of Cren7 in the standard assay using primer template P36/C72 (Figure 7A and B). RNA strand displacement by the polymerase was not affected by Cren7, as observed with Sso7d (Figure 7C and D). The presence of PCNA and RFC did not influence the ability of Cren7 to modulate strand displacement synthesis by PolB1 (Figure 7E and F). In addition, Cren7 also displayed lower affinity for the RNA:DNA hybrid than for the DNA duplex (Figure 8A and B) and stabilized the former less effectively than the latter (Figure 8C and D). These results show that the two chromatin proteins are similar in modulating strand displacement by PolB1.

However, differences were found between the two chromatin proteins in how they affect strand displacement by PolB1. First, the two proteins differed in concentration dependence in the inhibition of strand displacement. The lengths of the extension products decreased gradually with increasing amounts of Sso7d with maximal inhibition at a protein concentration of 25 μM, when the template was probably saturated by the protein as revealed by EMSA conducted in parallel (Figures 2A and 6A). By contrast, strand displacement was not significantly reduced by Cren7 at <0.16 μM but maximally inhibited when the protein concentration was raised to 0.32 μM (Figure 7A). Notably, Cren7 started to bind the template at concentrations below 0.16 μM and did not saturate the template at 0.32 μM (Figure 8A). Second, in the RNA/DNA chimera strand displacement assays, chain extension by PolB1 was blocked by Sso7d primarily after the first deoxyribonucleotide at the RNA-DNA junction was displaced, whereas displacement of at least two nucleotides into the downstream DNA sequence of the primer occurred when Sso7d was replaced by Cren7 (Figures 4C and 7D). It has been shown that, despite their similarity, Cren7 and Sso7d differ in DNA binding affinity, binding size and ability to constrain DNA supercoils (26). Therefore, it appears that Cren7 and Sso7d interact with DNA in different manners, and this provides a possible explanation for the differences between the two chromatin proteins in their influence on strand displacement by PolB1.

## DISCUSSION

DNA polymerases vary in strand displacement ability. In Bacteria, *Escherichia coli* Pol III, T4 and T7 DNA polymerases lack an intrinsic strand displacement activity (44–46), whereas phi 29 DNA polymerase from a bacteriophage in *Bacillus subtilis* is capable of synthesizing strand displacement products exceeding 70 kb (47). In Eukarya, Pol δ was shown to displace a DNA strand no longer than 72 nt (4). In Archaea, a family D DNA polymerase from *Pyrococcus abyssi* (PabPolD) is able to displace efficiently a 30-nt downstream strand (48). Here we show that PolB1, the main replicative DNA polymerase in *S. solfataricus*, was capable of displacing thousands of nucleotides at physiological temperatures. The difference between the two hyperthermophilic DNA polymerases in strand displacement efficiency at high temperature [(48) and unpublished data] and the observation that PolB1 remained active in strand displacement at lower temperatures (e.g. 37°C), when polymerization activity of the enzyme was drastically reduced, point to the intrinsic nature of the strand displacement activity of PolB1. Structural analysis demonstrates that PolB1 possesses an unusual fingers subdomain made up of four α helices instead of two as observed in other DNA polymerases (49). Because the α helices are known to play key roles in the separation of downstream DNA during strand displacement (50), PolB1 may be structurally adapted for its robust strand displacement activity.

Eukarya use two pathways in Okazaki fragment maturation, i.e. the short flap pathway and the long flap pathway, based on the length of the flap generated during the process (51). Strand displacement by a DNA polymerase plays a decisive role in pathway selection. The ability of PolB1 to displace an extra long DNA strand would necessitate the use of the long flap pathway by *S. solfataricus* in processing Okazaki fragments. However, Dna2, which is required for the eukaryal long flap pathway, is not present in *Sulfolobus* (24). Furthermore, uncontrolled generation of extra long flaps *in vivo* would create an unbearable burden for the organism in DNA replication with respect to the energy expenses. In this study, we show that *S. solfataricus* has evolved a mechanism, involving the use of chromatin proteins, for the control of strand displacement by PolB1. *Sulfolobus* encodes two structurally similar chromatin proteins, Sso7d and Cren7. Both proteins inhibited DNA strand displacement by PolB1, whether it was alone or in association with PCNA. A DNA flap of only 3–4 nt in size was generated by PolB1 when the template was bound to saturation by either protein. One possible explanation for the mechanism of the inhibition is that the intrinsic 5′–3′ polymerization of PolB1 was directly inhibited by the chromatin proteins. This apparently was not the case because gap-filling synthesis was not inhibited in the presence of the chromatin proteins. In fact, Sul7d was reported to enhance chain extension by PolB1 at high temperatures (38). Another explanation is that the chromatin proteins inhibited PolB1-mediated strand displacement by increasing the stability of dsDNA. This is consistent with the ability of the chromatin proteins to raise the melting temperature of dsDNA. A recent single-molecule study showed that forces assisting template opening strongly stimulates the strand displacement activity of DNA polymerase (52). Conceivably, binding by the chromatin proteins prevents template opening, thereby inhibiting strand displacement by PolB1.

PolB1 was capable of displacing 3–4 nt of the downstream strand even when the primer template was saturated by Sso7d or Cren7. The mechanism of the invasion of 3–4 nt into the downstream strand by PolB1 is unclear, but this property of the polymerase appears to be well adapted for Okazaki fragment maturation. In Eukarya, RNase H serves to remove RNA primers (3). Although RNase H is believed to function primarily in DNA repair in Archaea (53,54), the possibility exists that the enzyme also plays a role in Okazaki fragment maturation. RNase H may cleave much of an RNA primer, leaving behind the last ribonucleotide upstream of the RNA–DNA junction, which is then removed by the 5′–3′ exonuclease activity of Fen1 (55). However, the efficiency of the removal of the last ribonucleotide was lower than that of flap cleavage by Fen1 (18). We speculate that strand displacement may occur following RNA primer degradation by RNase H. The ability of PolB1 to displace 3–4 nt into a downstream base-paired primer in the presence of the chromatin proteins allows the last ribonucleotide to be displaced and cleaved efficiently.

Archaeal Okazaki fragments are typically primed by an RNA primer of ∼10 nt in length (56). Neither Sso7d nor Cren7 inhibited displacement of an RNA strand from a DNA template. When a 5′-RNA-DNA chimera primer was used, strand displacement was mostly blocked at the first or the second deoxyribonucleotide next to the junction between the RNA and the DNA stretches by Sso7d or Cren7, respectively. Displacement of 1–2 deoxyribonucleotides downstream of the RNA–DNA junction may be due to less efficient binding by the chromatin protein to the end than to the rest of the DNA stretch. The apparent selectivity in strand displacement permits removal of an RNA primer synthesized by low-fidelity primase while leaving behind the more accurately synthesized DNA on the lagging strand. Our results suggest that the differential effects of the chromatin proteins on DNA and RNA displacement from a DNA template may arise from the difference of the proteins in binding and stabilizing RNA:DNA and DNA:DNA duplexes. The RNA portion of an Okazaki fragment, readily displaced by PolB1 during lagging strand synthesis, will be removed during the subsequent maturation step. Therefore, we conclude that the chromatin proteins may play two roles in Okazaki fragment maturation in *S. solfataricus*. First, the chromatin proteins inhibit DNA strand displacement by PolB1, allowing Okazaki fragments to be processed in a short flap pathway. Second, they show differential effects on DNA and RNA strand displacement, allowing ready removal of RNA primers in Okazaki fragments by Fen1. Intriguingly, Sso7d and Cren7 appear to behave differently in modulating strand displacement by PolB1. Cren7 exhibited maximal inhibition of strand displacement at a protein concentration lower than that required to saturate the primer template, while Sso7d showed the highest inhibition of strand displacement only when the primer template was saturated by the protein. In addition, Cren7 maximally inhibited DNA strand displacement at much lower concentration (0.32 μM) than Sso7d (∼25 μM). Furthermore, PolB1 was able to displace mostly a single nucleotide and at least two nucleotides when it ploughed into the DNA portion of a 5′-RNA-DNA chimera primer annealed to a DNA template in the presence of Sso7d and Cren7, respectively, when the protein existed at a saturating level. It appears that Cren7 is a more efficient and more reliable modulator of strand displacement by PolB1 than Sso7d.

In Eukarya, RPA binds DNA flaps of >30 nt and inhibits Fen1 cleavage (9). *Sulfolobus solfataricus* lacks RPA homologs, but encodes monomeric single-stranded DNA binding proteins (SSB), which have a binding size of 5 nt (57). In the presence of saturating amounts of a chromatin protein, DNA flaps of only 3–4 nt was generated. Presumably, these flaps will not be bound stably by SSB and, thus, will be readily cleaved by Fen1. Given the intracellular Sso7d/DNA mass ratio of ∼4 (25), or a protein molecule for 2.5 base pairs, and the binding size of 4 bp for the protein, Sso7d alone is sufficiently abundant to coat the entire genome in *Sulfolobus* cells. On the other hand, Cren7 is less abundant than Sso7d in the cell (26), with one protein molecule for every 12.5 base pairs. Since it has a binding size of 8 bp (26), Cren7 is able to cover two thirds of the genomic DNA *in vivo*. Based on the above calculation, we speculate that Okazaki fragments are likely saturated by the chromatin proteins and other DNA-binding proteins, and DNA flaps longer than 5 nt may not be readily generated *in vivo*. Consistent with this suggestion is the finding that Okazaki fragment processing in *S. solfataricus* includes multiple rounds of flap creation and cleavage, and the majority of the cleavage products were 1–4 nt in size (23), suggesting that SSB is probably not involved in flap processing.

**Figure 9.**
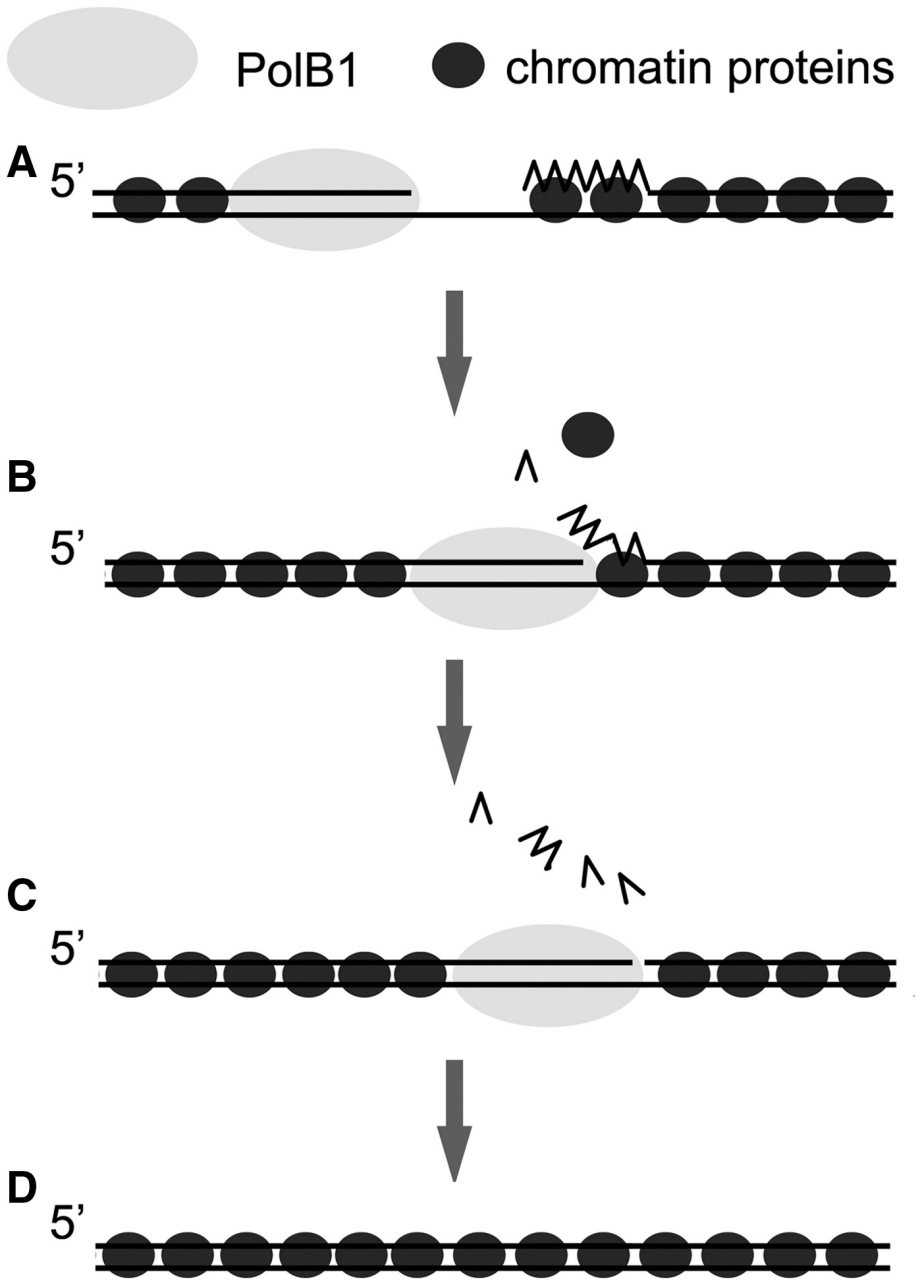
A model for the role of *Sulfolobus* chromatin proteins in Okazaki fragment maturation. Chromatin proteins, bound to double-stranded regions during lagging strand synthesis (**A**), become disassociated from the RNA:DNA hybrid region when PolB1 displaces the RNA primer (**B**). The displaced strand undergoes multiple rounds of flap cleavage by Fen1. PolB1 stops strand displacement on entering the dsDNA region, which is stably bound by the chromatin proteins (**C**). The resulting nick was sealed by DNA ligase as the newly synthesized Okazaki fragment was ligated into the lagging strand (**D**). PCNA, Fen1 and DNA ligase are omitted.

Based on our results, we propose the following model for the roles of the chromatin proteins in Okazaki fragment maturation in *S. solfataricus* (Figure 9). Once an Okazaki fragment is synthesized on the lagging strand, Sso7d and Cren7 preferentially bind and stabilize the dsDNA region of the fragment. The RNA:DNA hybrid portion is also bound by the proteins with lower binding affinities. When a polymerizing PolB1 molecule on the lagging strand encounters the 5′-end of a downstream primer, it starts displacing the primer, and the chromatin proteins became disassociated from the region. The RNA primer was entirely displaced and removed, probably by cycles of flap creation and Fen1 cleavage. Once PolB1 enters the dsDNA region, which are tightly bound by the chromatin proteins, strand displacement is blocked, and the gap is subsequently sealed.

Of the two main lineages in Archaea, Crenarchaea and Euryarchaea use Cren7/Sul7d- and histone-based chromatin packaging strategies, respectively. Like Cren7 and Sso7d, euryarchaeal histones are also capable of similar inhibition of strand displacement (unpublished results). Moreover, the function of histones in terminating Okazaki fragment synthesis and avoiding excessive strand displacement by Pol δ in *Saccharomyces cerevisiae* has been reported (58). Therefore, it appears that, despite their evolutionary differences, the chromatin proteins from different organisms share similar functions not only in chromosomal packaging but also in DNA replication.

## SUPPLEMENTARY DATA

Supplementary Data are available at NAR Online.

## FUNDING

National Natural Science Foundation of China (NSFC) [31130003 and 30921065]. Funding for open access charge: NSFC [31130003].

*Conflict of interest statement*. None declared.

## Supplementary Material

Supplementary Data

## References

[1] Pursell ZF, Isoz I, Lundstrom EB, Johansson E, Kunkel TA (2007). Yeast DNA polymerase epsilon participates in leading-strand DNA replication. Science.

[2] Nick McElhinny SA, Gordenin DA, Stith CM, Burgers PM, Kunkel TA (2008). Division of labor at the eukaryotic replication fork. Mol. Cell.

[3] Zheng L, Shen B (2011). Okazaki fragment maturation: nucleases take centre stage. J. Mol. Cell Biol..

[4] Maga G, Villani G, Tillement V, Stucki M, Locatelli GA, Frouin I, Spadari S, Hubscher U (2001). Okazaki fragment processing: modulation of the strand displacement activity of DNA polymerase delta by the concerted action of replication protein A, proliferating cell nuclear antigen, and flap endonuclease-1. Proc. Natl Acad. Sci. USA.

[5] Rossi ML, Bambara RA (2006). Reconstituted Okazaki fragment processing indicates two pathways of primer removal. J. Biol. Chem..

[6] Tom S, Henricksen LA, Bambara RA (2000). Mechanism whereby proliferating cell nuclear antigen stimulates flap endonuclease 1. J. Biol. Chem..

[7] Ayyagari R, Gomes XV, Gordenin DA, Burgers PM (2003). Okazaki fragment maturation in yeast. I. Distribution of functions between FEN1 and Dna2. J. Biol. Chem..

[8] Bochkareva E, Korolev S, Lees-Miller SP, Bochkarev A (2002). Structure of the RPA trimerization core and its role in the multistep DNA-binding mechanism of RPA. EMBO J..

[9] Bae SH, Bae KH, Kim JA, Seo YS (2001). RPA governs endonuclease switching during processing of Okazaki fragments in eukaryotes. Nature.

[10] Stewart JA, Miller AS, Campbell JL, Bambara RA (2008). Dynamic removal of replication protein A by Dna2 facilitates primer cleavage during Okazaki fragment processing in *Saccharomyces cerevisiae*. J. Biol. Chem..

[11] Gloor JW, Balakrishnan L, Campbell JL, Bambara RA (2012). Biochemical analyses indicate that binding and cleavage specificities define the ordered processing of human Okazaki fragments by Dna2 and FEN1. Nucleic Acids Res..

[12] Dionne I, Nookala RK, Jackson SP, Doherty AJ, Bell SD (2003). A heterotrimeric PCNA in the hyperthermophilic archaeon *Sulfolobus solfataricus*. Mol. Cell.

[13] Dore AS, Kilkenny ML, Jones SA, Oliver AW, Roe SM, Bell SD, Pearl LH (2006). Structure of an archaeal PCNA1-PCNA2-FEN1 complex: elucidating PCNA subunit and client enzyme specificity. Nucleic Acids Res..

[14] Lai X, Shao H, Hao F, Huang L (2002). Biochemical characterization of an ATP-dependent DNA ligase from the hyperthermophilic crenarchaeon *Sulfolobus shibatae*. Extremophiles.

[15] Pascal JM, Tsodikov OV, Hura GL, Song W, Cotner EA, Classen S, Tomkinson AE, Tainer JA, Ellenberger T (2006). A flexible interface between DNA ligase and PCNA supports conformational switching and efficient ligation of DNA. Mol. Cell.

[16] Hosfield DJ, Frank G, Weng Y, Tainer JA, Shen B (1998). Newly discovered archaebacterial flap endonucleases show a structure-specific mechanism for DNA substrate binding and catalysis resembling human flap endonuclease-1. J. Biol. Chem..

[17] Mase T, Kubota K, Miyazono K, Kawarabayasi Y, Tanokura M (2011). Structure of flap endonuclease 1 from the hyperthermophilic archaeon *Desulfurococcus amylolyticus*. Acta Crystallogr. Sect. F Struct. Biol. Cryst. Commun..

[18] Matsui E, Kawasaki S, Ishida H, Ishikawa K, Kosugi Y, Kikuchi H, Kawarabayashi Y, Matsui I (1999). Thermostable flap endonuclease from the archaeon, *Pyrococcus horikoshii*, cleaves the replication fork-like structure endo/exonucleolytically. J. Biol. Chem..

[19] Imamura K, Fukunaga K, Kawarabayasi Y, Ishino Y (2007). Specific interactions of three proliferating cell nuclear antigens with replication-related proteins in *Aeropyrum pernix*. Mol. Microbiol..

[20] Keppetipola N, Shuman S (2005). Characterization of a thermophilic ATP-dependent DNA ligase from the euryarchaeon *Pyrococcus horikoshii*. J. Bacteriol..

[21] Rogozin IB, Makarova KS, Pavlov YI, Koonin EV (2008). A highly conserved family of inactivated archaeal B family DNA polymerases. Biol. Direct.

[22] Choi JY, Eoff RL, Pence MG, Wang J, Martin MV, Kim EJ, Folkmann LM, Guengerich FP (2011). Roles of the four DNA polymerases of the crenarchaeon *Sulfolobus solfataricus* and accessory proteins in DNA replication. J. Biol. Chem..

[23] Beattie TR, Bell SD (2012). Coordination of multiple enzyme activities by a single PCNA in archaeal Okazaki fragment maturation. EMBO J..

[24] Beattie TR, Bell SD (2011). The role of the DNA sliding clamp in Okazaki fragment maturation in archaea and eukaryotes. Biochem. Soc. Trans..

[25] Mai VQ, Chen X, Hong R, Huang L (1998). Small abundant DNA binding proteins from the thermoacidophilic archaeon *Sulfolobus shibatae* constrain negative DNA supercoils. J. Bacteriol..

[26] Guo L, Feng Y, Zhang Z, Yao H, Luo Y, Wang J, Huang L (2007). Biochemical and structural characterization of Cren7, a novel chromatin protein conserved among Crenarchaea. Nucleic Acids Res..

[27] Zhang Z, Gong Y, Guo L, Jiang T, Huang L (2010). Structural insights into the interaction of the crenarchaeal chromatin protein Cren7 with DNA. Mol. Microbiol..

[28] Choli T, Henning P, Wittmann-Liebold B, Reinhardt R (1988). Isolation, characterization and microsequence analysis of a small basic methylated DNA-binding protein from the Archaebacterium, *Sulfolobus solfataricus*. Biochim. Biophys. Acta.

[29] Baumann H, Knapp S, Lundback T, Ladenstein R, Hard T (1994). Solution structure and DNA-binding properties of a thermostable protein from the archaeon *Sulfolobus solfataricus*. Nat. Struct. Biol..

[30] McAfee JG, Edmondson SP, Zegar I, Shriver JW (1996). Equilibrium DNA binding of Sac7d protein from the hyperthermophile *Sulfolobus acidocaldarius*: fluorescence and circular dichroism studies. Biochemistry.

[31] Lundback T, Hansson H, Knapp S, Ladenstein R, Hard T (1998). Thermodynamic characterization of non-sequence-specific DNA-binding by the Sso7d protein from *Sulfolobus solfataricus*. J. Mol. Biol..

[32] Peters WB, Edmondson SP, Shriver JW (2004). Thermodynamics of DNA binding and distortion by the hyperthermophile chromatin protein Sac7d. J. Mol. Biol..

[33] McAfee JG, Edmondson SP, Datta PK, Shriver JW, Gupta R (1995). Gene cloning, expression, and characterization of the Sac7 proteins from the hyperthermophile *Sulfolobus acidocaldarius*. Biochemistry.

[34] Napoli A, Zivanovic Y, Bocs C, Buhler C, Rossi M, Forterre P, Ciaramella M (2002). DNA bending, compaction and negative supercoiling by the architectural protein Sso7d of *Sulfolobus solfataricus*. Nucleic Acids Res..

[35] Lopez-Garcia P, Knapp S, Ladenstein R, Forterre P (1998). *In vitro* DNA binding of the archaeal protein Sso7d induces negative supercoiling at temperatures typical for thermophilic growth. Nucleic Acids Res..

[36] Driessen RP, Meng H, Suresh G, Shahapure R, Lanzani G, Priyakumar UD, White MF, Schiessel H, van Noort J, Dame RT (2013). Crenarchaeal chromatin proteins Cren7 and Sul7 compact DNA by inducing rigid bends. Nucleic Acids Res..

[37] Feng Y, Yao H, Wang J (2010). Crystal structure of the crenarchaeal conserved chromatin protein Cren7 and double-stranded DNA complex. Protein Sci..

[38] Lou H, Duan Z, Huo X, Huang L (2004). Modulation of hyperthermophilic DNA polymerase activity by archaeal chromatin proteins. J. Biol. Chem..

[39] Lowry OH, Rosebrough NJ, Farr AL, Randall RJ (1951). Protein measurement with the Folin phenol reagent. J. Biol. Chem..

[40] Zuo Z, Lin HK, Trakselis MA (2011). Strand annealing and terminal transferase activities of a B-family DNA polymerase. Biochemistry.

[41] Lou H, Duan Z, Sun T, Huang L (2004). Cleavage of double-stranded DNA by the intrinsic 3′-5′ exonuclease activity of DNA polymerase B1 from the hyperthermophilic archaeon *Sulfolobus solfataricus* at high temperature. FEMS Microbiol. Lett..

[42] Jin YH, Ayyagari R, Resnick MA, Gordenin DA, Burgers PM (2003). Okazaki fragment maturation in yeast. II. Cooperation between the polymerase and 3′-5′-exonuclease activities of Pol delta in the creation of a ligatable nick. J. Biol. Chem..

[43] Garg P, Burgers PM (2005). How the cell deals with DNA nicks. Cell Cycle.

[44] O'Donnell ME, Kornberg A (1985). Complete replication of templates by *Escherichia coli* DNA polymerase III holoenzyme. J. Biol. Chem..

[45] Hacker KJ, Alberts BM (1994). The rapid dissociation of the T4 DNA polymerase holoenzyme when stopped by a DNA hairpin helix. A model for polymerase release following the termination of each Okazaki fragment. J. Biol. Chem..

[46] Ghosh S, Marintcheva B, Takahashi M, Richardson CC (2009). C-terminal phenylalanine of bacteriophage T7 single-stranded DNA-binding protein is essential for strand displacement synthesis by T7 DNA polymerase at a nick in DNA. J. Biol. Chem..

[47] Blanco L, Bernad A, Lazaro JM, Martin G, Garmendia C, Salas M (1989). Highly efficient DNA synthesis by the phage phi 29 DNA polymerase. Symmetrical mode of DNA replication. J. Biol. Chem..

[48] Henneke G, Flament D, Hübscher U, Querellou J, Raffin JP (2005). The hyperthermophilic euryarchaeota *Pyrococcus abyssi* likely requires the two DNA polymerases D and B for DNA replication. J. Mol. Biol..

[49] Savino C, Federici L, Johnson KA, Vallone B, Nastopoulos V, Rossi M, Pisani FM, Tsernoglou D (2004). Insights into DNA replication: the crystal structure of DNA polymerase B1 from the archaeon *Sulfolobus solfataricus*. Structure.

[50] Singh K, Srivastava A, Patel SS, Modak MJ (2007). Participation of the fingers subdomain of *Escherichia coli* DNA polymerase I in the strand displacement synthesis of DNA. J. Biol. Chem..

[51] Balakrishnan L, Bambara RA (2011). Eukaryotic lagging strand DNA replication employs a multi-pathway mechanism that protects genome integrity. J. Biol. Chem..

[52] Manosas M, Spiering MM, Ding F, Bensimon D, Allemand JF, Benkovic SJ, Croquette V (2012). Mechanism of strand displacement synthesis by DNA replicative polymerases. Nucleic Acids Res..

[53] Mesiet-Cladiere L, Norais C, Kuhn J, Briffotaux J, Sloostra JW, Ferrari E, Hubscher U, Flament D, Myllykallio H (2007). A novel proteomic approach identifies new interaction partners for proliferating cell nuclear antigen. J. Mol. Biol..

[54] Bubeck D, Reijns MA, Graham SC, Astell KR, Jones EY, Jackson AP (2011). PCNA directs type 2 RNase H activity on DNA replication and repair substrates. Nucleic Acids Res..

[55] Turchi JJ, Huang L, Murante RS, Kim Y, Bambara RA (1994). Enzymatic completion of mammalian lagging-strand DNA replication. Proc. Natl Acad. Sci. USA.

[56] Matsunaga F, Norais C, Forterre P, Myllykallio H (2003). Identification of short ‘eukaryotic' Okazaki fragments synthesized from a prokaryotic replication origin. EMBO Rep..

[57] Wadsworth RI, White MF (2001). Identification and properties of the crenarchaeal single-stranded DNA binding protein from *Sulfolobus solfataricus*. Nucleic Acids Res..

[58] Smith DJ, Whitehouse I (2012). Intrinsic coupling of lagging-strand synthesis to chromatin assembly. Nature.

