# Supplementary material for: Sulfolobus chromatin proteins modulate strand displacement by DNA polymerase B1
Source: Nucleic Acids Res. 2013 Jul 1;41(17):8182–95. doi: 10.1093/nar/gkt588 (PMC3783171; doi:10.1093/nar/gkt588)
Supplement: Supplementary Data [file supp_41_17_8182__index.html]

Sulfolobus chromatin proteins modulate strand displacement by DNA polymerase B1 — Sulfolobus chromatin proteins modulate strand displacement by DNA polymerase B1 — Supplementary Data 

# *Sulfolobus* chromatin proteins modulate strand displacement by DNA polymerase B1

## 

files

**Files in this Data Supplement:**

- Supplementary Data - pdf file
