# Supplementary material for: Sulfolobus chromatin proteins modulate strand displacement by DNA polymerase B1
Source: Nucleic Acids Res. 2013 Jul 1;41(17):8182–95. doi: 10.1093/nar/gkt588 (PMC3783171; doi:10.1093/nar/gkt588)
Supplement: Supplementary Data [file supp_gkt588_nar-00368-m-2013-File011.pdf]

Table S1. Oligonucleotides used in this study.

Oligo name	Sequence
L72	5'-CTTCTAGTTGTGAATTCGGCACTGGCCGTCGTATGCTCTTGGTTGTA GGATCCCAGCACATTGAAGGATGCA
Bridger	5'-CAGTGCCGAATTCACAACTAGAAGTGCATCCTTCAATGT
P17	5'-TGCATCCTTCAATGTGC
P36	5'-TGCATCCTTCAATGTGCTGGGATCCTACAACCAAGA
P59	5'-TGCATCCTTCAATGTGCTGGGATCCTACAACCAAGAGCATACGACG GCCAGTGCCGAAT
P36(5'AT-rich)	5'-TTCAATGTGCTGGGATCCTACAACCAAGAGCATACG
P36(5'GC-rich)	5'-GCTGGGATCCTACAACCAAGAGCATACGACGGCCAG
P36(flap5)	5'-TTTTTTGCATCCTTCAATGTGCTGGGATCCTACAACCAAGA
P36(flap10)	5'-TTTTCTTTTTTGCATCCTTCAATGTGCTGGGATCCTACAACCAAGA
P36(5'RNA)	5'-*UGCAUCCUUCAATGTGCTGGGATCCTACAACCAAGA
D30	5'-TGCTTCCTTCAATGTGCTGGGATCCTACAA
R30	5'-UGCUUCCUUCAAUGUGCUGGGAUCCUACAA
Com30	5'-TTGTAGGATCCCAGCACATTGAAGGAAGCA
D15	5'-TGCATCCTTCAAGAC
R15	5'-UGCAUCCUUCAAGAC
Com15	5'-GTCTTGAAGGATGCA

*ribonucleotides are underlined.
